# Outbreak of SARS-CoV-2: challenge for diagnosis and medical management in patients with left ventricular assist device: a case series

**DOI:** 10.1093/ehjcr/ytaa447

**Published:** 2021-03-07

**Authors:** Pan Dan, Frotscher Birgit, Mathieu Matei, Veronique Vuillemin, Helene Ottenin, Pablo-Juan Maureira, Fabrice Vanhuyse

**Affiliations:** 1 Department of Cardiovascular Surgery and Transplantation, Regional Central Hospital of Lorraine University, Rue de Morvan, 54500 Nancy, France; 2 Department of Laboratory of Hematology, Hemostasis Unit, Regional Central Hospital of Lorraine University, Rue de Morvan, 54500 Nancy, France; 3 Department of Intensive Care Unit of Cardiac Surgery, Regional Central Hospital of Lorraine University, Rue de Morvan, 54500 Nancy, France

**Keywords:** Case series, COVID-19, Left ventricular assist device, LVAD

## Abstract

**Background:**

The outbreak of coronavirus disease 2019 (COVID-19) exposes vulnerable patients to high risk of mortality. Patients with left ventricular assist device (LVAD) usually have symptoms such as cough, fever, and shortness of breath because of their cardiac condition and comorbidity, therefore these related symptoms challenge the correct diagnosis in time within the COVID-19 pandemic.

**Case summary:**

We report two case studies of patients with LVAD in whom COVID-19 related symptoms were overlapped by their cardiac status and comorbidities. In the first case, the patient was admitted for suspicion of COVID-19 due to cough and shortness of breath for 1 month. The blood test evocated a high index of suspicion of COVID-19. The nasopharyngeal test for COVID-19 performed on admission and at Day 2 was inconclusive for severe acute respiratory syndrome coronavirus 2 (SARS-CoV-2), but the test obtained on Day 3 of admission was positive, whereas computed tomography confirmed the diagnosis of COVID-19. This patient developed acute respiratory distress syndrome (ARDS) and nasal epistaxis within 48 h during hospitalization. The ARDS was treated by non-invasive ventilation and probabilistic antibiotics for 3 days and resulted significant improvement. The nasal epistaxis due to international normalized ratio increase was treated by nasal packing and vitamin K antagonist was switched to parenteral heparin infusion. The patient was kept hospitalized for 1 month for further supportive treatment. In the second case, the patient was admitted for recurrent anaemia due to melaena, the patient was tested for COVID-19 because of new-onset symptoms of cough and rhinorrhoea. The first nasopharyngeal test was positive, and sudden increase of anticoagulation status was noted in the setting of gastrointestinal bleeding. The anticoagulation status was controlled by parenteral heparin infusion, and the melaena was disappeared at Day 3. The moderate dyspnoea of the patient was quickly improved with nasal oxygen delivery for 4 days. The patient was discharged at Day 5.

**Discussion:**

COVID-19 specific symptoms are challenging to distinguish in patients with LVADs, although radiological evidence can be beneficial in the COVID-19 diagnosis. We also observed the need for precise anticoagulation control to avoid bleeding or thrombotic events in these patients.

**Learning points**

- Left ventricular assist device (LVAD) patients need careful examination to distinguish coronavirus disease 2019 (COVID-19) related symptoms that can be masked by patients’ underlying cardiac condition and comorbidities.
- For patients with LVAD and suspected of COVID-19, radiological tests are indispensable in cases of negative nasopharyngeal tests.
- Vitamin K antagonist should be replaced by parenteral heparin in COVID-19 positive patients with LVAD, and anti-factor Xa activity rather than active partial thromboplastin time, should be taken into consideration for anticoagulation monitoring.

## Introduction

The outbreak of coronavirus disease 2019 (COVID-19), illness caused by severe acute respiratory syndrome coronavirus 2 (SARS-CoV-2) infection, has caused more than 294 190 deaths worldwide by 14 May 2020.^1^ Elderly people and patients with certain medical conditions are particularly vulnerable in this pandemic. COVID-19 was shown to be responsible for various organ injuries and disorders, including cardiac injury which is associated with higher risk of mortality.^2^ Patients with left ventricular assist devices (LVAD) are known to have advanced heart failure and are usually associated with numerous comorbidities such as chronic obstructive pulmonary disease (COPD), diabetes mellitus, renal failure, gastrointestinal bleeding, and LVAD driveline infections.^3^ These patients can suffer constantly from diverse symptoms such as cough and shortness of breath due to their poor cardiac condition, and sometimes a fever due to local driveline infection.^4^ The symptoms of a patient with advanced heart failure are often similar to symptoms of COVID-19 which may be challenging for physicians to take the right steps for differential diagnosis and could potentially delay both the diagnosis and appropriate medical treatment. LVAD patients need regular dressing changes at the driveline exit site, which is often performed by a self-employed nurse. This continuous contact can put these patients at a higher risk of exposure of SARS-CoV-2. This report describes two cases of patients with LVAD that were infected by SARS-CoV-2, their diagnostic course, impact of COVID-19 on their anticoagulation status and the patients’ overall medical management.

## Timeline

**Figure:**
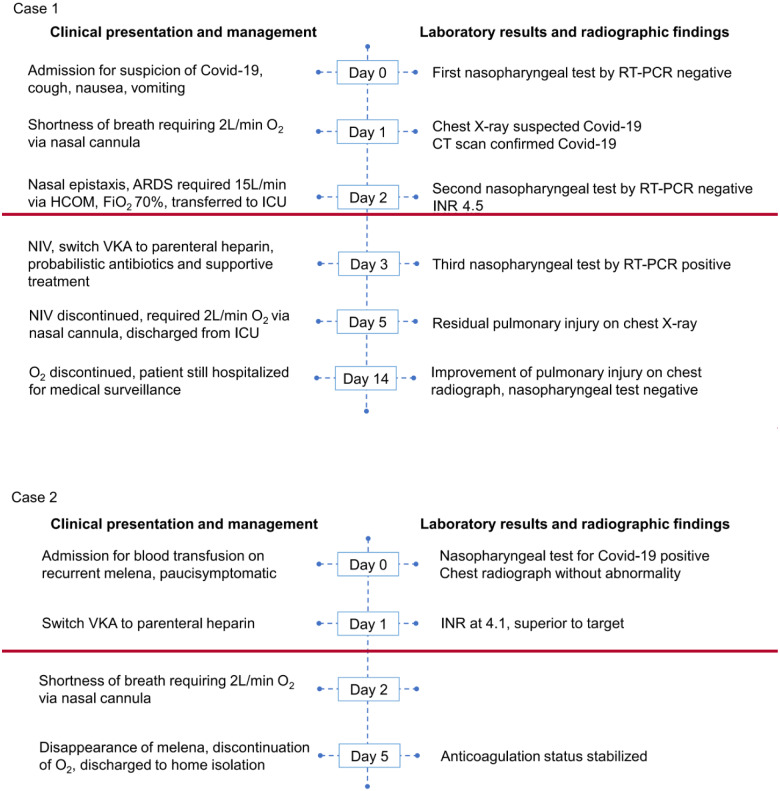


## Case presentation

### Case 1

**Figure 1:**
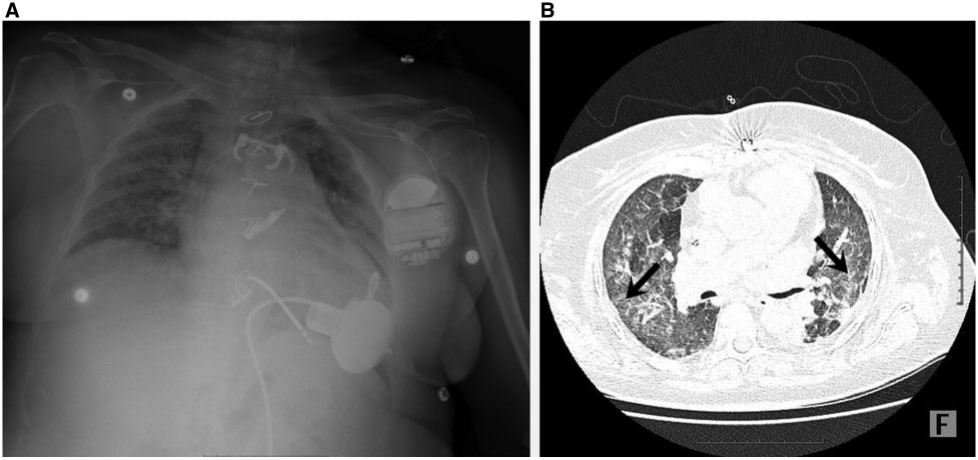
Radiological findings in the left ventricular assist device patient with positive COVID-19. (*A*) Chest X-ray demonstrated diffused bilateral opacities. (*B*) Computed tomography scan showed bilateral ground glass opacities and pulmonary consolidation.

The first case concerned a 74-year-old women with a LVAD (HeartWare, Medtronic^®^) implanted in 2017 because of ischaemic myocardiopathy. This patient is also known to have implantable cardioverter-defibrillator, chronic renal failure, diabetes mellitus, morbid obesity (body mass index 32.89 kg/m^2^), obstructive arteriopathy of the lower limbs, and chronic infection on the driveline exit site under control with oral antibiotics. The patient described a cough and shortness of breath for 1 month on a scheduled teleconsultation for her LVAD on 27 March 2020. Following the teleconsultation, the patient was admitted immediately to the department of cardiac surgery for suspicion of COVID-19. The patient was admitted to a single-patient room with contact, droplet, and airborne precautions, including eye protection. On admission, the physical examination revealed normal parameters (mean arterial blood pressure of 75 mmHg, 36.8°C, respiratory rate at 23 breaths per minute, 95% saturation on ambient air). Pulmonary auscultation revealed bilateral end-respiratory cackles, cardiac auscultation in the context of LVAD pump did not reveal other distinguishable murmur. The patient was thus authorized to take her usual medications including oral vitamin K antagonist (VKA). The blood test evocated a high index of suspicion of COVID-19. The nasopharyngeal swab test for COVID-19 was performed on hospital Day 0 by real-time reverse transcriptase–polymerase chain reaction (RT-PCR) and the result was negative. The patient became dyspnoeic on hospital Day 1 warranting oxygen delivered by nasal cannula at 2 L/min. A chest radiograph was performed on Day 1, which was reported as showing bilateral consolidative pulmonary opacity (*Figure 1A*). A computed tomography (CT) was then performed which confirmed the diagnosis of COVID-19 (*Figure 1B*).

On Day 2, the patient rapidly developed acute respiratory distress syndrome (ARDS), which was treated with high concentration oxygen (FiO_2_ 70%) delivered by mask at 15 L/min to maintain her saturation at 92%. The patient’s respiratory rate increased from 24 to 32 breaths per minute within 4 h. Meanwhile, the patient presented with significant nasal epistaxis that required nasal packing. The patient’s international normalized ratio (INR) was noted at 4.5 after the epistaxis started, which was an increase from her previous day’s INR which was 3.0 with her usual dose of VKA. Other haemostatic markers were also found to be abnormal (*Table 1*). Given the changing clinical presentation and the patient requiring more advanced care, the patient was transferred to the intensive care unit. A second nasopharyngeal test was performed in order to have nucleic acid confirmation of COVID-19, which also came back negative. The patient was treated by non-invasive ventilation for her hypoxia (FiO_2_ 70%). The VKA was switched to parenteral heparin infusion in order to better control the anticoagulation status. For the pneumonia based on clinical presentation and radiologic findings, treatment with cefotaxime (2 g, intravenous infusion, t.i.d.), spiramycin (1 g, per Os, t.i.d.) and oseltamivir (75 mg, per Os, q.d.) was initiated on hospital Day 2. Nevertheless, a third test for COVID-19 was performed on hospital Day 3, and it was positive for COVID-19. On Day 5, the patient’s clinical condition improved, non-invasive ventilation was discontinued, and supplemental oxygen was decreased back to 2 L/min delivered via nasal cannula with an oxygen saturation of 98%. The patient was then transferred to the department of infectious disease.

**Figure 2:**
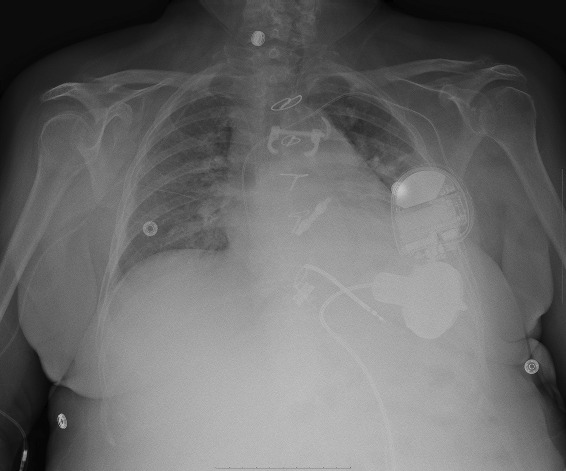
Chest X-ray on Day 28 showing decreased pulmonary opacities.

In the following days, the patient developed acute renal failure related to intensive antibiotic use and was treated accordingly (evolutionary laboratory results in *Table 1*). On Day 14, another nasopharyngeal PCR was performed and the results reported back as negative, and a chest X-ray demonstrated decreased ground glass opacity (*Figure 2*). The patient was asymptomatic apart from her cough, which was decreasing in severity. During the patient’s time in the hospital, her anticoagulation was monitored with regular anti-factor Xa activity (Anti-Xa) measurements and no bleeding or thrombotic event were observed following the nasal epistaxis on Day 3. However, the antihaemophilia factor A (factor VIII) and Von Willebrand factor remained elevated (*Table 1*), which reflects the inflammatory and prothrombotic status. The LVAD function remained stable during this hospitalization without any alarms. The patient remains hospitalized at the time of this report (1 month) and has improved physical tolerance.

### Case 2

**Figure 3:**
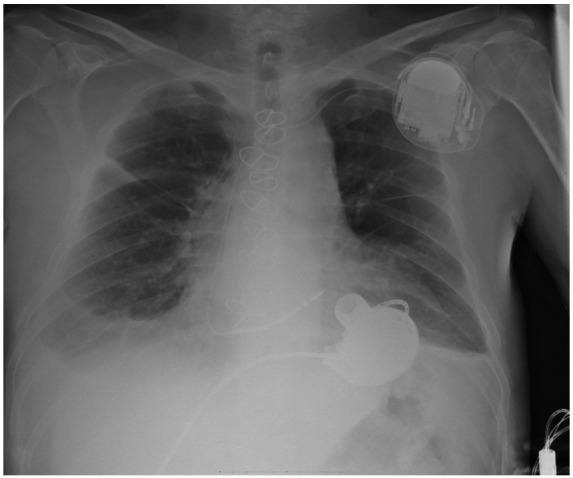
Chest radiograph on Day 0 did not show pulmonary opacity. Residual pleural effusion was due to the patient’s last pleural drainage.

A 64-year-old man was admitted to our department for blood transfusion due to melaena from ongoing gastrointestinal bleeding on 20 March 2020. This patient was implanted with an LVAD (HeartWare, Medtronic^®^) in 2012 because of ischaemic myocardiopathy and has constant shortness of breath due to COPD and recurrent anaemia due to gastrointestinal bleeding. Several previous endoscopic procedures have revealed arteriovenous malformations. The patient previously had two periods of LVAD thrombosis with subtherapeutic INR inferior to 2. On admission, the physical examination did not reveal any respiratory issues. The patient continued to take his usual medications except oral VKA. The patient stated new-onset symptoms of cough and rhinorrhoea for approximately 1 week. With these new-onset symptoms, a nasopharyngeal swab test for COVID-19 was performed which confirmed COVID-19 by a positive result on the following day. Because the exact onset of his COVID-19 course could not be determined, we decided to keep the patient in hospital for respiratory surveillance after the blood transfusion for his gastrointestinal bleed. A chest radiograph taken on Day 0 did not show opacity signs (*Figure 3*). The patient did not have fever during his hospital admission, and blood tests did not show any inflammatory signs. Supplemental oxygen was initiated on Day 2 because the oxygen saturation dipped to 90%, with oxygen delivery via nasal cannula at 1 L/min. Oxygen was discontinued on the morning of hospital Day 5.

The patient’s INR on admission was at 4.1, whereas his last INR (1 week prior) was at 3.2. VKA was stopped and parenteral heparin was initiated on Day 0. A total of 3 units red blood cells was transfused which restored haemoglobin from 7.7 to 10 g/dL. The melaena decreased progressively with precise control of anti-Xa under heparin infusion, with complete resolution on hospital Day 3. With the melaena resolved, VKA was reintroduced on hospital Day 3. During this hospitalization, the LVAD parameters remained stable without any alarms.

With the improvement of haemoglobin and resolution of melaena, as well as patient-reported resolution of shortness of breath, he was discharged home. As part of the discharge planning the patient remained in isolation with regular teleconsultation to better control his INR using a Coagucheck^®^ INRange system, and also continue to monitor respiratory parameters. We have scheduled a video-capsule gastrointestinal exam for the patient after the pandemic crisis resolves. The patient remains asymptomatic to date, although no chest radiograph in follow-up was performed in order to reduce transmission risk during the quarantined period.

## Discussion

**Figure 4:**
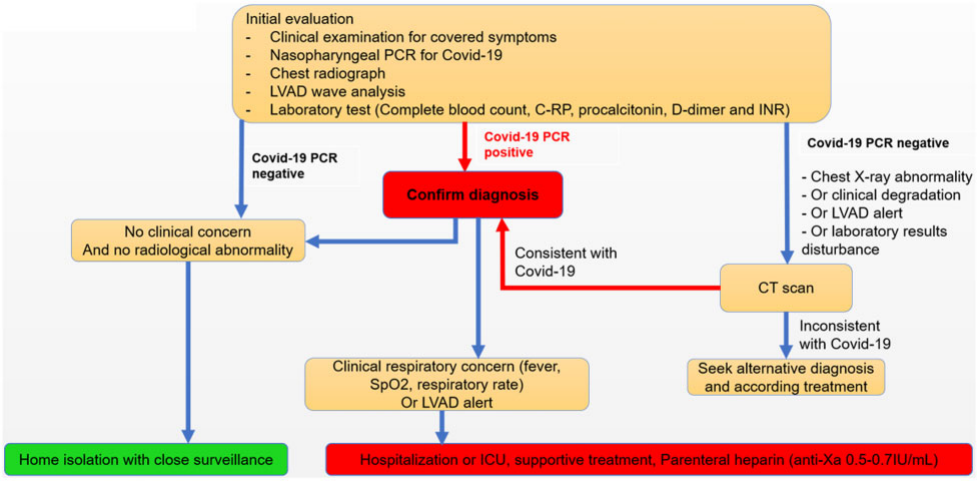
Medical management of left ventricular assist device patients during the COVID-19 pandemic. Once a patient is suspected to be infected by COVID-19, a nasopharyngeal swab should be tested by RT-PCR, and a chest radiograph should be taken at the same time. If the RT-PCR is positive, the COVID-19 diagnosis is made. If the RT-PCR is negative, the diagnosis should depend on clinical presentation and the chest radiograph or computed tomography scan. Whatever the RT-PCR result is, the medical management depends on the patient’s clinical presentation and radiological findings. For mild symptoms without radiological abnormality, patient can be followed remotely under home isolation with close surveillance. For moderate to severe clinical presentation with typical radiological changes on computed tomography scan, patients should be hospitalized to receive supportive treatment and VKA should be replaced by parenteral heparin, which should be monitored by anti-Xa targeting 0.5–0.7 IU/mL for effective anticoagulation.

Our report of two cases of patients with LVADs and infected by COVID-19 illustrates several aspects of the influence of the COVID-19 pandemic on a specific population of vulnerable people. *Figure 4* presents our proposed management strategy for LVAD patients.

For LVAD patients, more regular teleconsultation is needed to discover COVID-19 related symptoms. The two patients in our case report both presented with new-onset symptoms related to COVID-19, but diagnosis was complicated as both patients were used to having these COVID-19 related symptoms as part of their underlying heart failure. In the case of the first patient, she stated to have a cough for 1 month, but she reported this new-onset symptom only on a regularly scheduled teleconsultation. The second patient was tested for COVID-19 because of shortness of breath that, although often present during his anaemic periods, was reported by the patient as accompanied by rhinorrhoea for 1 week prior. These epidemiologic histories indicate that patients with an LVAD can be less vigilant of COVID-19 since it is difficult for them to discover specific COVID-19 related symptoms due to their underlying cardiac condition-related symptoms. As SARS-CoV-2 infection can rapidly progress to ARDS and lead to poor outcomes,^5^ correct and timely diagnosis can be vital for patients with LVAD. We suggest that physicians caring for LVAD patients during this pandemic period should organize more frequent teleconsultations to find possible early SARS-CoV-2 infections in these patients.

The impact of COVID-19 on LVAD patients can vary from mild respiratory symptoms to COVID-19 related ARDS. Our second patient presented with only a mild cough and rhinorrhoea related to COVID-19 and remained relatively stable throughout hospitalization. However, our first patient rapidly developed COVID-19 related ARDS within 4 h of presentation. The diagnosis and medical management were quite challenging for patient number 1 because of the first two negative nasopharyngeal COVID-19 tests. Current nasopharyngeal tests are reporting non-negligible false negative rates due to the quality of the swab, therefore CT images are now accepted as one of the diagnostic criteria of COVID-19,^6^^,^^7^ and its value was clearly proven in our patient. Therefore, we suggest that in LVAD patients with suspicion of COVID-19, radiological exams should be systematically performed. In cases of negative nasopharyngeal tests, these exams can help to confirm the diagnosis. Furthermore, chest radiographs can guide the medical management of LVAD patients with COVID-19. If a patient presents with mild respiratory symptoms and radiological exams reveal no related concerns, patients can be monitored at home with active contact with the healthcare centre. Otherwise, LVAD patients with COVID-related radiological changes should be hospitalized to quickly react to potential sudden degradation.

LVADs need well-controlled anticoagulation to prevent thrombosis, which could otherwise result in life-threatening dysfunction of the device. On the other hand, LVAD patients have higher risk of haemorrhage events due to anticoagulation therapy. It was reported that COVID-19 can alter the patients’ coagulation status, which can cause both thrombosis and haemorrhage.^8^ We observed active bleeding in our two patients with sudden increase in their INR values that were outside the target range. Our first patient presented with epistaxis requiring gauze packing. The second patient developed gastrointestinal bleeding and melaena, and while these symptoms have been previously seen in this patient, we were concerned that the instability of the INR may have been triggered by COVID-19. Thus, careful attention in anticoagulation status must be undertaken in LVAD patients. Parenteral heparin replacement can be used as an alternative to avoid additional bleeding or ischaemic events. We suggest hospitalizing LVAD patients with a COVID-19 diagnosis to receive parenteral heparin infusion until stabilization of clinical presentation. COVID-19 patients commonly have an inflammatory reaction and diverse circulatory antibodies which may disturb active partial thromboplastin time (aPTT),^9^ therefore Anti-Xa targeting between 0.5 and 0.7 IU/mL, rather than aPTT, should be taken into consideration for effective monitoring. In our opinion, in LVAD patients, if the active bleeding is life threatening, parenteral heparin should be stopped. In case of non-life threatening active bleeding, we propose an anti-Xa level between 0.3 and 0.5. While the active bleeding is controllable (in Case 1 the bleeding was controlled by nasal packing) or recurring chronic context (for example as observed in our Case 2), the anti-Xa level should be maintained between 0.5 and 0.7 to avoid LVAD thrombosis. In all cases, the underlying cause of active bleeding should be treated.

## Conclusion

This report highlights the need to closely monitor patients with LVADs during this COVID-19 pandemic, as the correct diagnosis is challenging due to their underlying cardiac disease and associated symptoms. Radiological exams are indispensable in LVAD patients with suspected or confirmed COVID-19 diagnosis since they can guide the medical management. In cases of patients with LVAD where the INR is deranged in the context of COVID-19 infection, it may be appropriate to covert from a VKA to parenteral heparin infusion to better control anticoagulation and avoid thrombotic and bleeding events.

## Lead author biography

**Figure:**
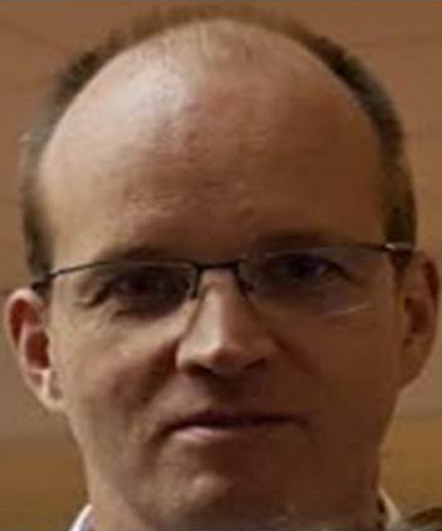


Fabrice Vanhuyse, MD, PhD is a cardiac surgeon and Director of the VAD Program.

## Supplementary material

Supplementary material is available at *European Heart Journal – Case Reports* online.

**Slide sets:** A fully edited slide set detailing this case and suitable for local presentation is available online as Supplementary data.

**Consent:** The author/s confirm that written consent for submission and publication of this case series including image(s) and associated text has been obtained from the patient in line with COPE guidance.

**Conflict of interest:** none declared.

**Funding:** The authors would like to thank the European Society of Cardiology for financially supporting this research.

## Supplementary Material

ytaa447_Supplementary_Data
