# Supplementary material for: Outbreak of SARS-CoV-2: challenge for diagnosis and medical management in patients with left ventricular assist device: a case series
Source: Eur Heart J Case Rep. 2021 Mar 7;5(3):ytaa447. doi: 10.1093/ehjcr/ytaa447 (PMC7946800; doi:10.1093/ehjcr/ytaa447)
Supplement: ytaa447_Supplementary_Data [file ytaa447_supplementary_data.pptx]

## Slide 1
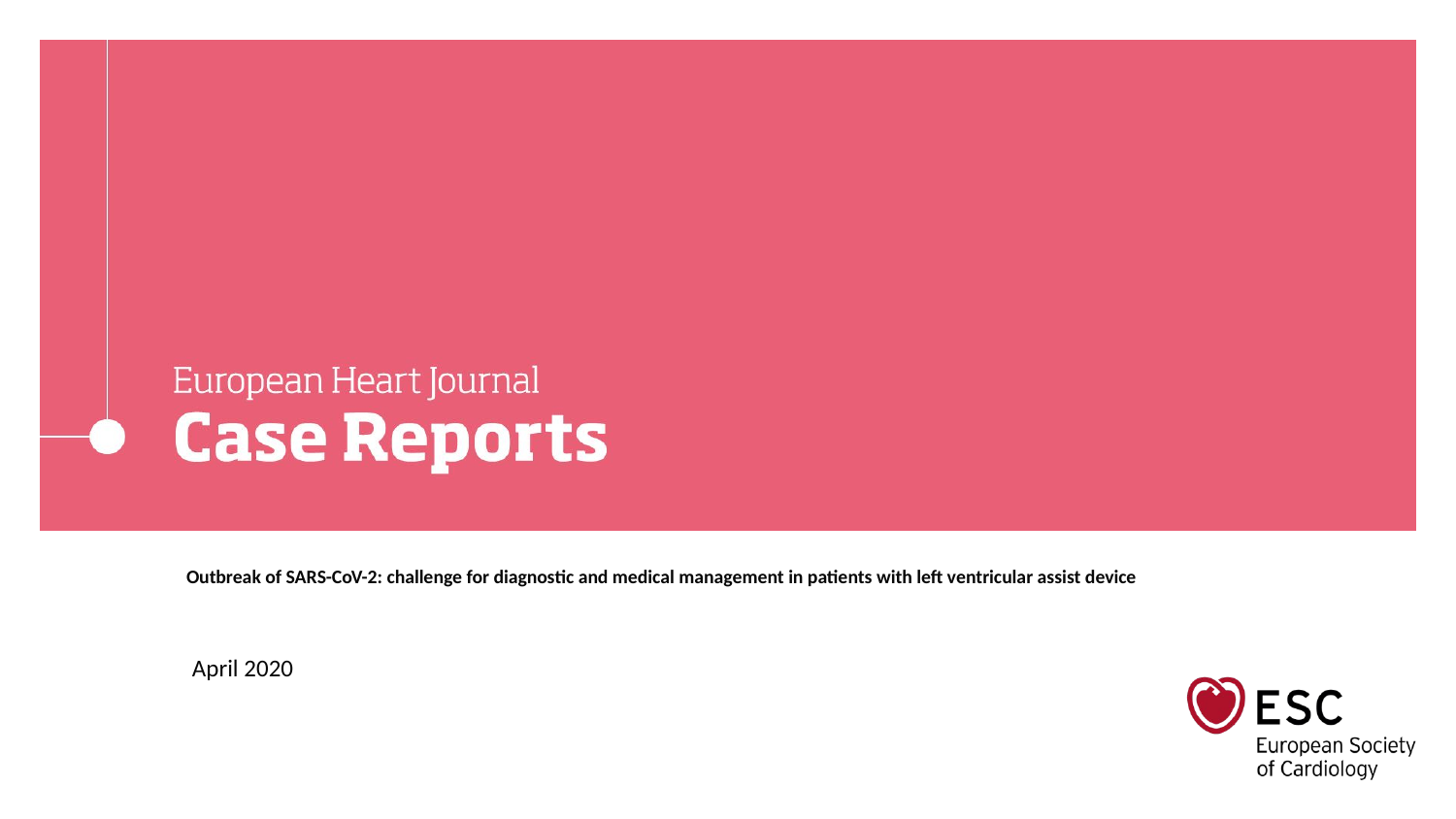

# Outbreak of SARS-CoV-2: challenge for diagnostic and medical management in patients with left ventricular assist device
April 2020

## Slide 2
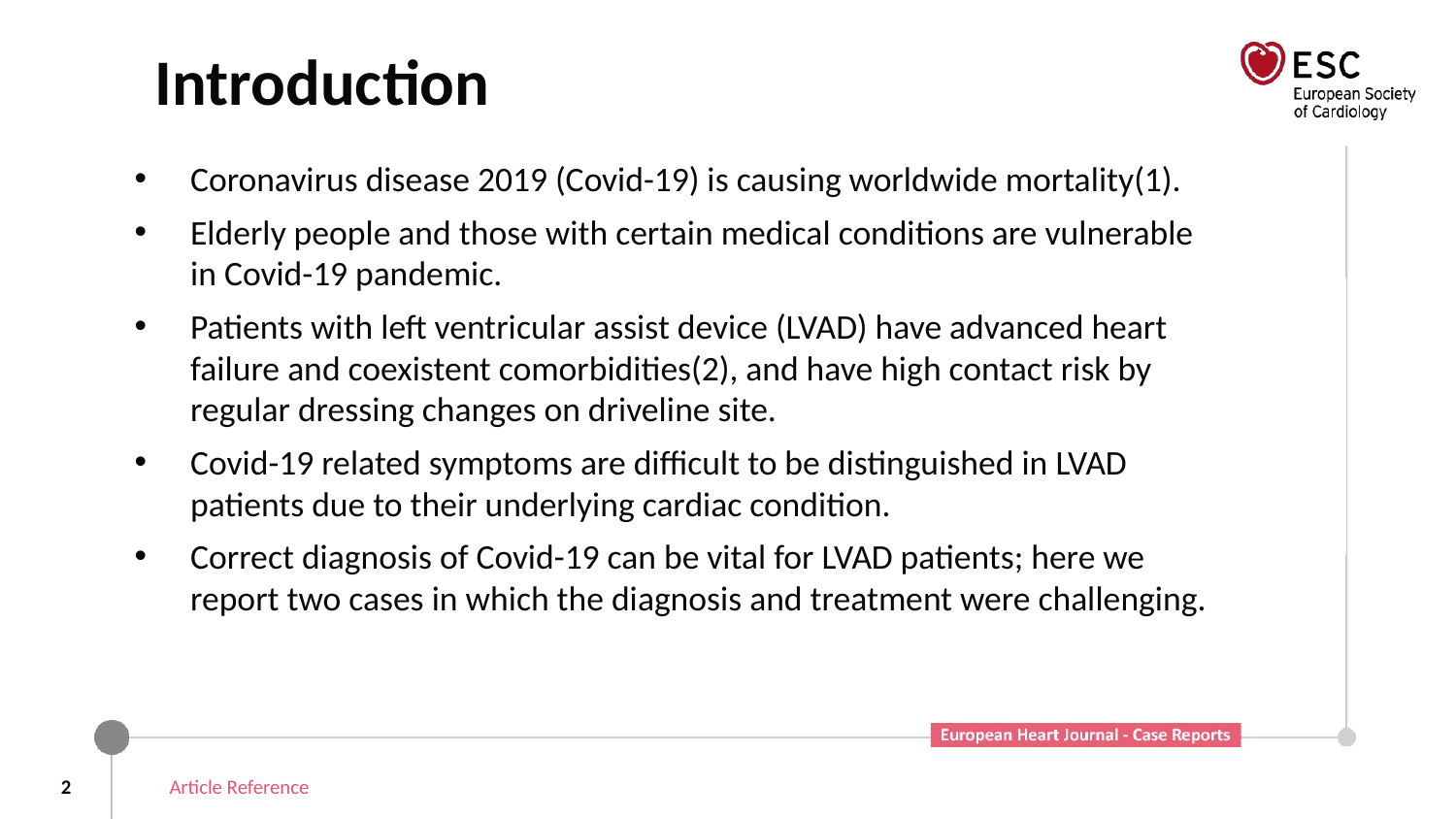

# Introduction
Coronavirus disease 2019 (Covid-19) is causing worldwide mortality(1).
Elderly people and those with certain medical conditions are vulnerable in Covid-19 pandemic.
Patients with left ventricular assist device (LVAD) have advanced heart failure and coexistent comorbidities(2), and have high contact risk by regular dressing changes on driveline site.
Covid-19 related symptoms are difficult to be distinguished in LVAD patients due to their underlying cardiac condition.
Correct diagnosis of Covid-19 can be vital for LVAD patients; here we report two cases in which the diagnosis and treatment were challenging.
2
Article Reference

## Slide 3
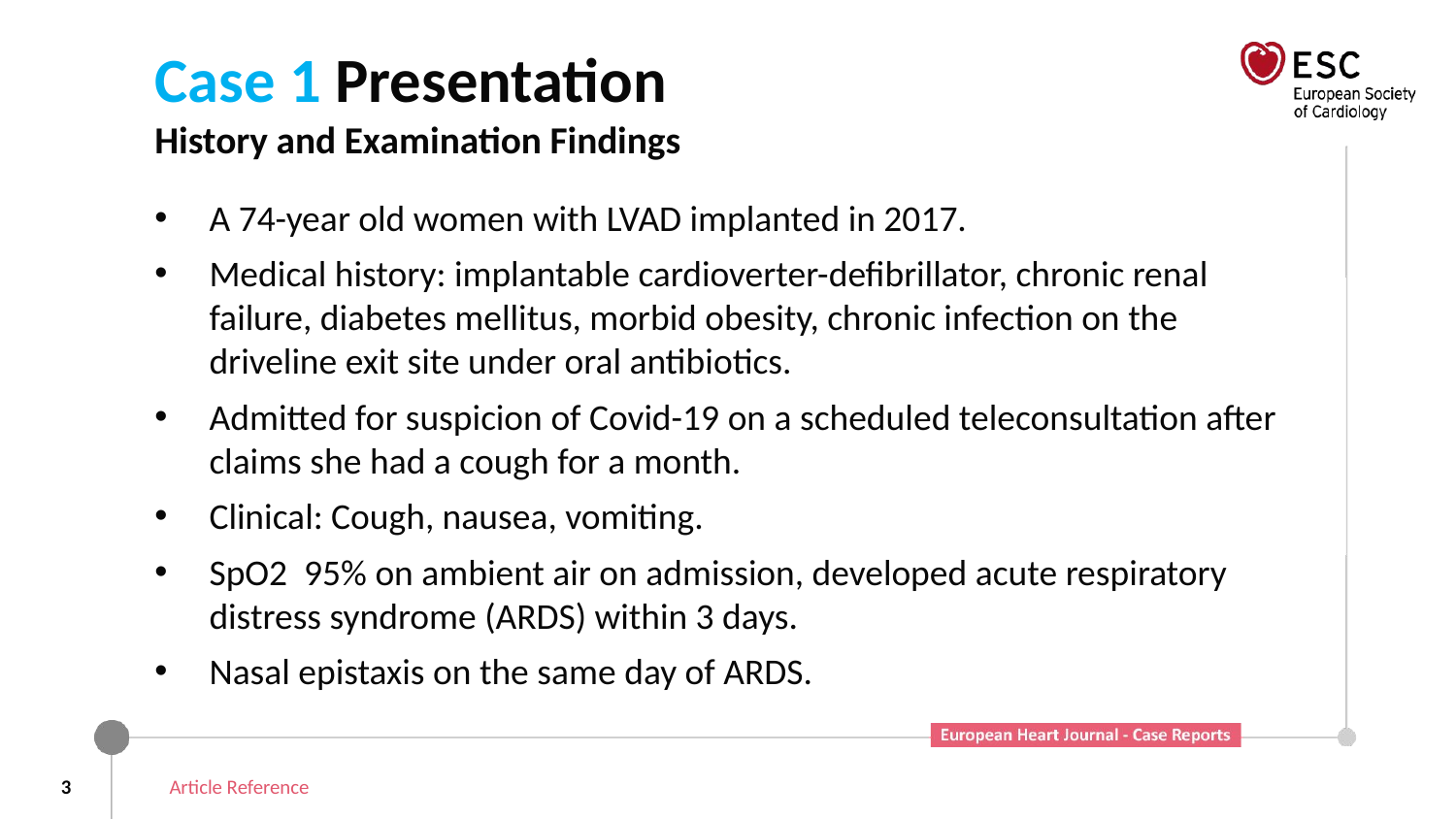

# Case 1 Presentation History and Examination Findings
A 74-year old women with LVAD implanted in 2017.
Medical history: implantable cardioverter-defibrillator, chronic renal failure, diabetes mellitus, morbid obesity, chronic infection on the driveline exit site under oral antibiotics.
Admitted for suspicion of Covid-19 on a scheduled teleconsultation after claims she had a cough for a month.
Clinical: Cough, nausea, vomiting.
SpO2 95% on ambient air on admission, developed acute respiratory distress syndrome (ARDS) within 3 days.
Nasal epistaxis on the same day of ARDS.
3
Article Reference

## Slide 4
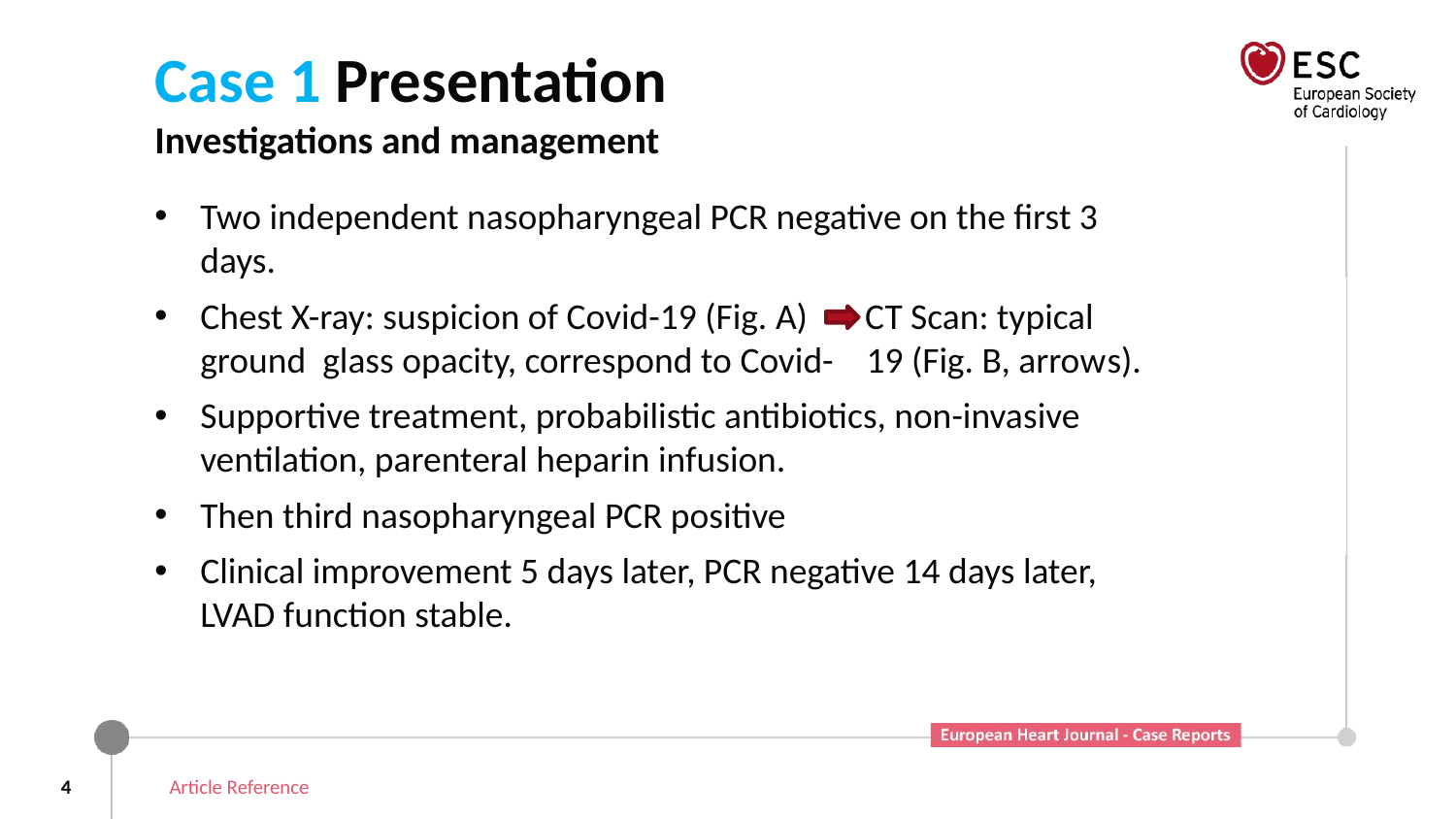

# Case 1 Presentation Investigations and management
Two independent nasopharyngeal PCR negative on the first 3 days.
Chest X-ray: suspicion of Covid-19 (Fig. A) CT Scan: typical ground glass opacity, correspond to Covid- 19 (Fig. B, arrows).
Supportive treatment, probabilistic antibiotics, non-invasive ventilation, parenteral heparin infusion.
Then third nasopharyngeal PCR positive
Clinical improvement 5 days later, PCR negative 14 days later, LVAD function stable.
4
Article Reference

## Slide 5
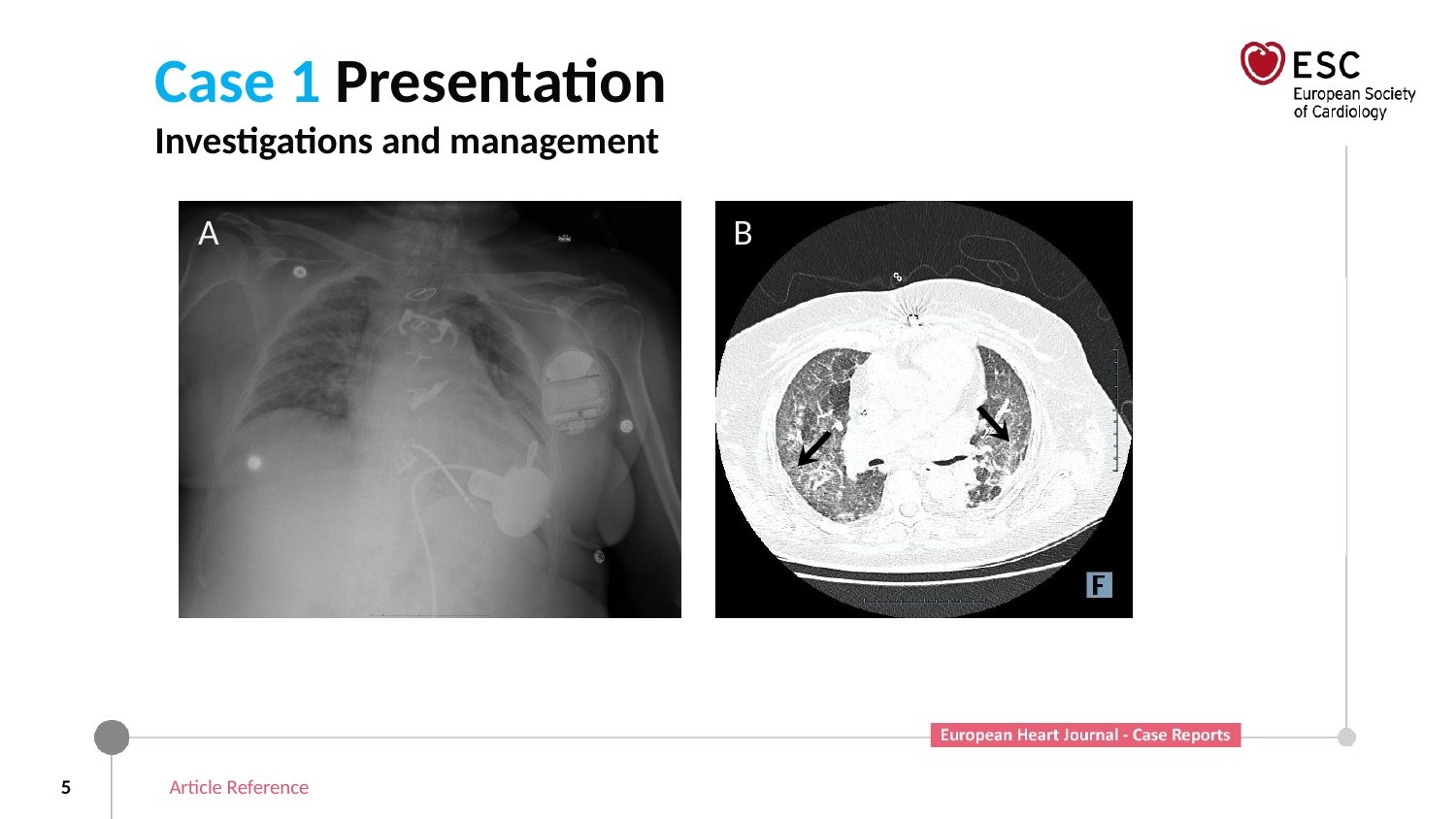

# Case 1 Presentation Investigations and management
A
B
5
Article Reference

## Slide 6
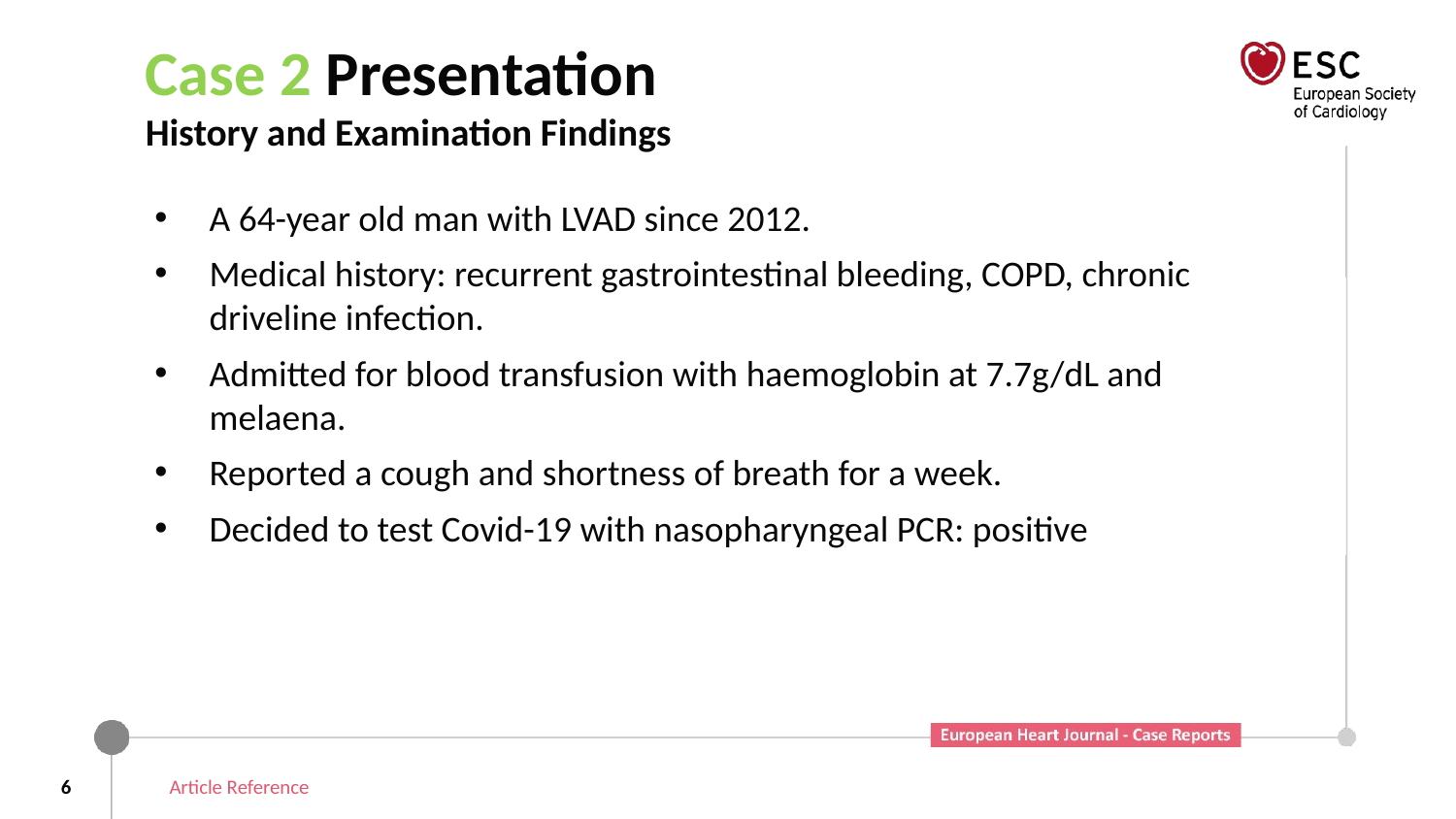

Case 2 Presentation
History and Examination Findings
A 64-year old man with LVAD since 2012.
Medical history: recurrent gastrointestinal bleeding, COPD, chronic driveline infection.
Admitted for blood transfusion with haemoglobin at 7.7g/dL and melaena.
Reported a cough and shortness of breath for a week.
Decided to test Covid-19 with nasopharyngeal PCR: positive
6
Article Reference

## Slide 7
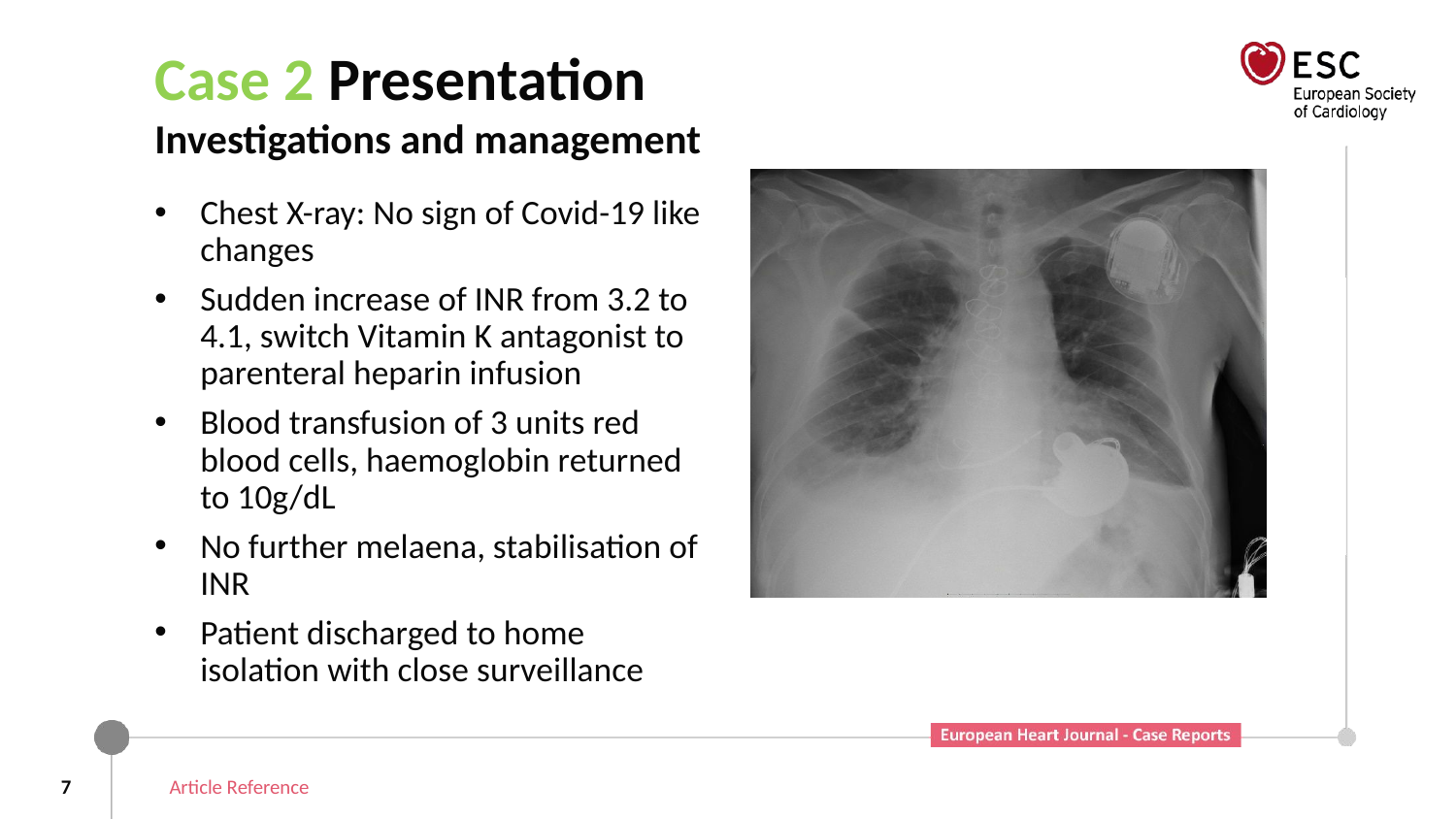

Case 2 Presentation
Investigations and management
Chest X-ray: No sign of Covid-19 like changes
Sudden increase of INR from 3.2 to 4.1, switch Vitamin K antagonist to parenteral heparin infusion
Blood transfusion of 3 units red blood cells, haemoglobin returned to 10g/dL
No further melaena, stabilisation of INR
Patient discharged to home isolation with close surveillance
7
Article Reference

## Slide 8
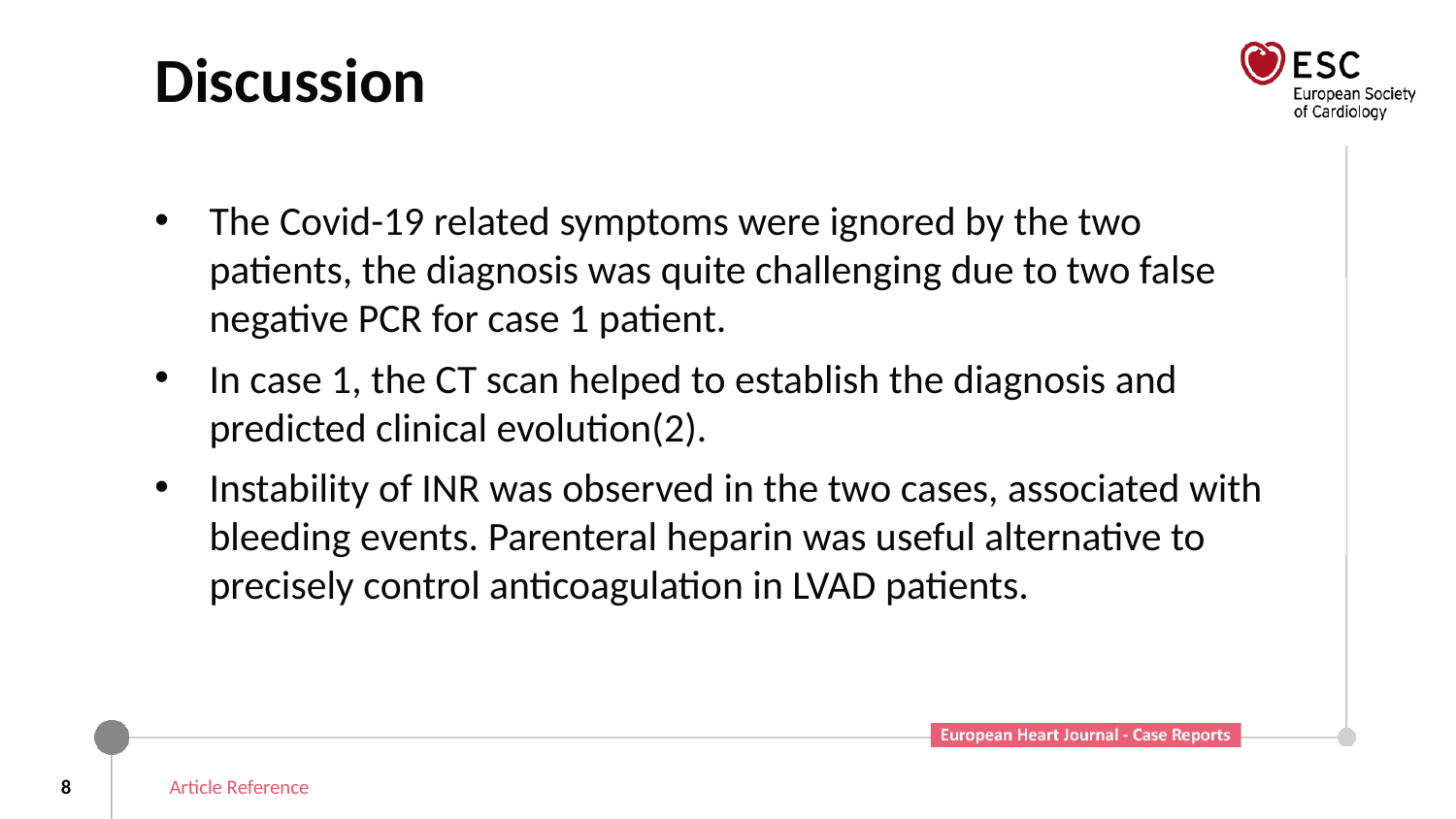

# Discussion
The Covid-19 related symptoms were ignored by the two patients, the diagnosis was quite challenging due to two false negative PCR for case 1 patient.
In case 1, the CT scan helped to establish the diagnosis and predicted clinical evolution(2).
Instability of INR was observed in the two cases, associated with bleeding events. Parenteral heparin was useful alternative to precisely control anticoagulation in LVAD patients.
8
Article Reference

## Slide 9
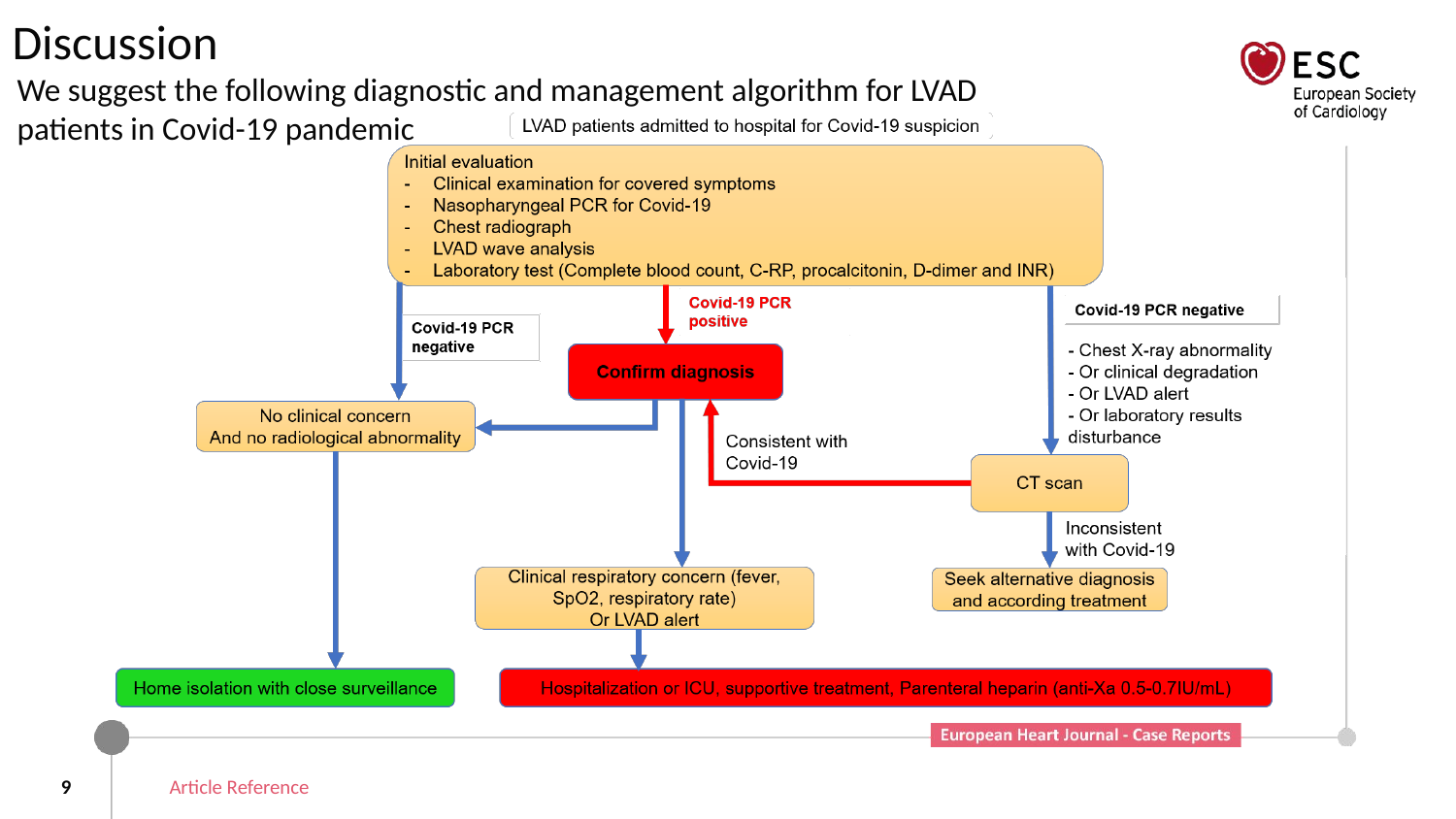

# Discussion
We suggest the following diagnostic and management algorithm for LVAD patients in Covid-19 pandemic
9
Article Reference

## Slide 10
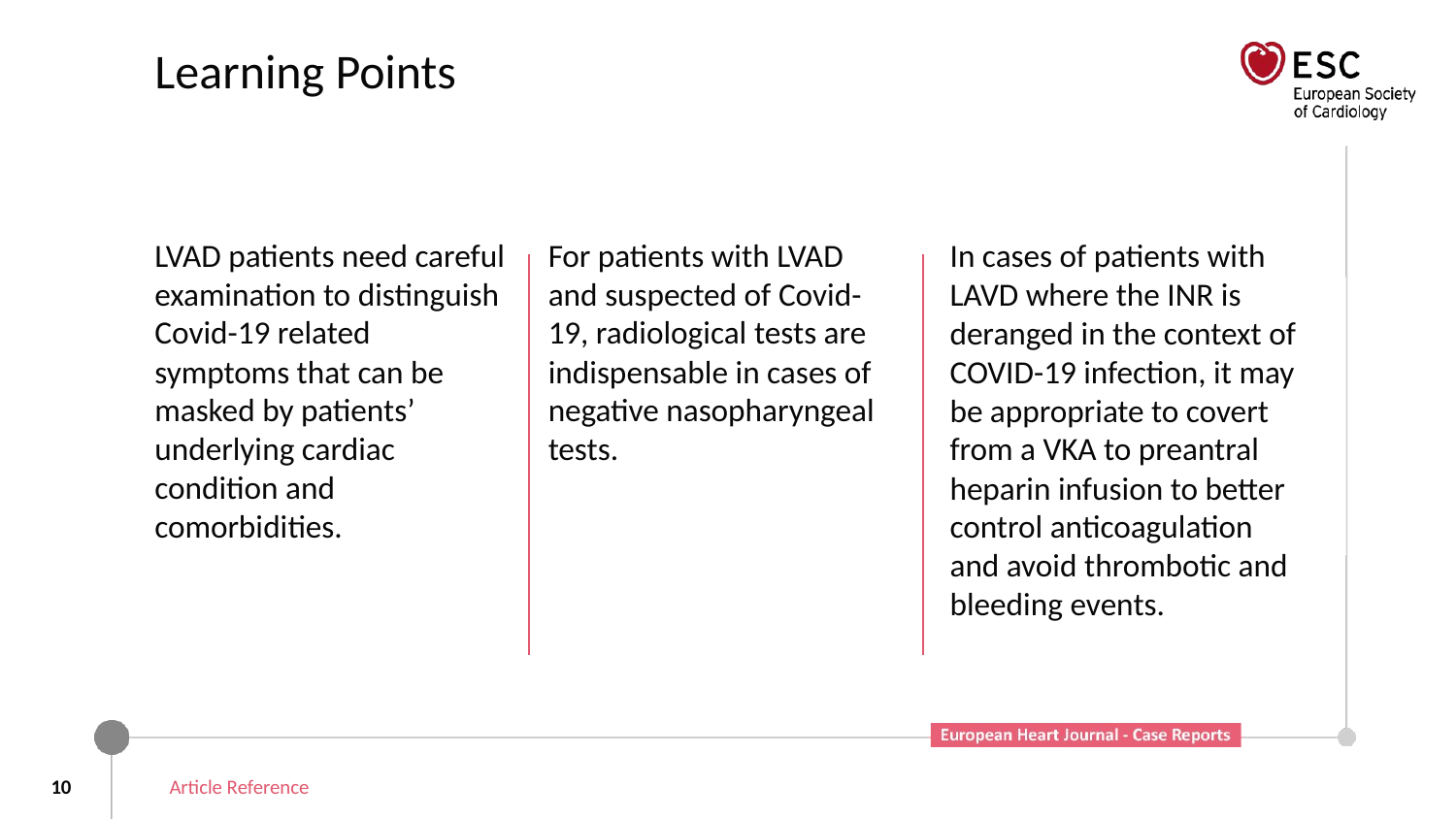

# Learning Points
LVAD patients need careful examination to distinguish Covid-19 related symptoms that can be masked by patients’ underlying cardiac condition and comorbidities.
For patients with LVAD and suspected of Covid-19, radiological tests are indispensable in cases of negative nasopharyngeal tests.
In cases of patients with LAVD where the INR is deranged in the context of COVID-19 infection, it may be appropriate to covert from a VKA to preantral heparin infusion to better control anticoagulation and avoid thrombotic and bleeding events.
10
Article Reference

## Slide 11
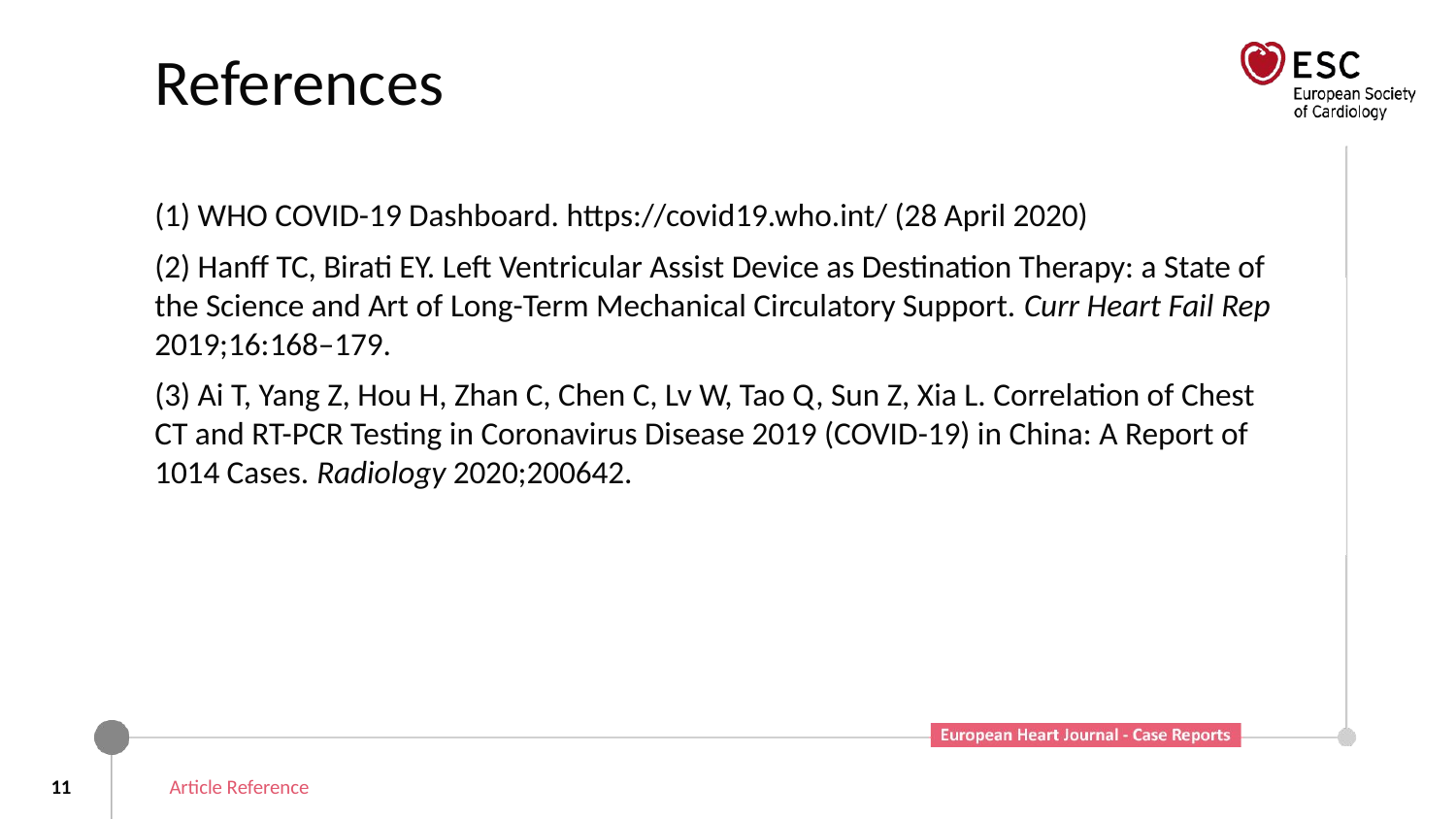

# References
(1) WHO COVID-19 Dashboard. https://covid19.who.int/ (28 April 2020)
(2) Hanff TC, Birati EY. Left Ventricular Assist Device as Destination Therapy: a State of the Science and Art of Long-Term Mechanical Circulatory Support. Curr Heart Fail Rep 2019;16:168–179.
(3) Ai T, Yang Z, Hou H, Zhan C, Chen C, Lv W, Tao Q, Sun Z, Xia L. Correlation of Chest CT and RT-PCR Testing in Coronavirus Disease 2019 (COVID-19) in China: A Report of 1014 Cases. Radiology 2020;200642.
11
Article Reference
